# Social representations of female sex workers about their sexuality

**DOI:** 10.17533/udea.iee.v38n1e03

**Published:** 2020-02-26

**Authors:** Pablo Luiz Santos Couto, Bianca Pereira Correia Montalvão, Arilene Rodrigues Silva Vieira, Alba Benemérita Alves Vilela, Sérgio Correia Marques, Antônio Marcos Tosoli Gomes, Núbia Rego Santos, Luiz Carlos Moraes França

**Affiliations:** 1 Nurse, Master. Professor, Higher Education Center of Guanambi, Brazil. Email: pablocouto0710@gmail.com Higher Education Center of Guanambi Brazil pablocouto0710@gmail.com; 2 Nurse, Specialist. Professor, Higher Education Center of Guanambi, Brazil. Email: biancapcm1@gmail.com Higher Education Center of Guanambi Brazil biancapcm1@gmail.com; 3 Nurse, Specialist. Professor, Higher Education Center of Guanambi, Brazil. Email: leneemariana@gmail.com Higher Education Center of Guanambi Brazil leneemariana@gmail.com; 4 Nurse, PhD. Professor, State University of Southwest Bahia, Brazil. Email: albavilela@gmail.com State University of Southwest Bahia Brazil albavilela@gmail.com; 5 Nurse, Doctor. Professor, State University of Rio de Janeiro, Brazil. Email: sergiocmarques@uol.com.br. Universidade Federal do Estado do Rio de Janeiro State University of Rio de Janeiro Brazil sergiocmarques@uol.com.br; 6 Nurse, Doctor. Professor, Rio de Janeiro State University, Brazil. Email: mtosoli@gmail.com Universidade Federal do Estado do Rio de Janeiro Rio de Janeiro State University Brazil mtosoli@gmail.com; 7 Nurse, Specialist. Professor, Higher Education Center of Guanambi, Brazil. Email: nubia.net12@gmail.com. Higher Education Center of Guanambi Brazil nubia.net12@gmail.com; 8 Nurse, Master. Professor, State University of Rio de Janeiro, Brazil. Email: lcmoraesfranca@hotmail.com Universidade Federal do Estado do Rio de Janeiro State University of Rio de Janeiro Brazil lcmoraesfranca@hotmail.com

**Keywords:** female, sex workers, pleasure, sexuality, sexual behavior, qualitative research., femenino, trabajadores sexuales, placer, sexualidad, conducta sexual, investigación cualitativa.

## Abstract

**Objective.:**

To know the social representations of female sex workers about their sexuality.

**Methods.:**

Qualitative study based on the Theory of Social Representations. Thirty-nine women from a health region of Alto Sertão Produtivo Baiano - Brazil agreed to participate. For the production of empirical data, the techniques of Free Word Association and in-depth interviews were used. The answers were analyzed based on Constellation Target Content Analysis and Semantic Content Analysis.

**Results.:**

Two thematic categories emerged: “negative representation of sexuality”; “my pleasure is the money”. Therefore, the theme sexuality and meanings derived from the social representations elaborated by the sex workers about sexuality, based on their experiences and daily life, showed that the work involved a negative representation of sexuality when associated with sexual satisfaction with the client, in addition to the allusion to sex as a source of income.

**Conclusion.:**

The social representations about sexuality constructed by sex workers are linked to the feeling of denial of pleasure and obtaining money for subsistence. Reflecting on sexuality points out ways to rethink the care to be provided for a stigmatized and vulnerable group.

## Resumen

**Objetivo.:**

Apreender las representaciones sociales de las trabajadoras sexuales sobre su sexualidad.

**Métodos.:**

Estudio cualitativo, basado en la Teoría de las Representaciones Sociales. Aceptaron participar 69 mujeres de una región productiva bahiana de salud en el Sertão (Brasil). Para la producción de los datos empíricos, se utilizaron las técnicas de Asociación Libre de Palabras y entrevistas a profundidad. Las respuestas se analizaron a partir del Análisis de Contenido por Objetivo de Constelación o de Contenido Semántico.

**Resultados.:**

Surgieron dos categorías temáticas: "Representación negativa de la sexualidad" y "Mi placer es el dinero". Para las trabajadoras sexuales, la sexualidad tiene una representación negativa cuando se asocia con la satisfacción sexual con el cliente, además de la alusión al sexo como fuente de ingreso económico.

**Conclusión.:**

Las representaciones sociales de la sexualidad construidas por las trabajadoras sexuales se refieren al sentimiento de negación del placer y a la obtención de dinero para la subsistencia. Reflexionar sobre la sexualidad aporta caminos para repensar el cuidado a un grupo estigmatizado por la sociedad y con alta vulnerabilidad social

## Introduction

The practice of prostitution has been a vulnerable work marginalized by society, because besides involving sexuality and human sexual practices in exchange for money, it is permeated by social stigmas. Women, the social actresses involved in sex work, find themselves inserted in a daily routine in which they resort to sexual practice as a service to offer in order to obtain profit and income for their own support and that of their families.(1) In this universe of sexual service, these workers set limits with clients on what is and/or is not allowed during sex, as a form of protection, as some of them support themselves in the sense of protection, since there is no guarantee on the part of the State of safety against violent situations and possible abuses that many clients tend to commit. Sex workers (a term used by the Ministry of Labor and Employment for prostitutes) offer a service that provides them autonomy and financial independence, as well as the satisfaction of personal and family needs.(1-3) 

Due to the social contexts in which they work and the subjectivity that is the product of affection and culture, sex workers are inserted into the group of vulnerable populations by various social reasons. For example, they use sex as service and are susceptible to Sexually Transmitted Infections (STIs). Also, they circulate and work in a diversity of spaces, including bars, brothels, hotels, squares, streets and avenues, places that do not offer security.(4) However, these workers object the way society inserts them into these vulnerable groups, because most of them protect themselves, care for themselves and prevent STIs.(5) What they claim is the protection of the State, recognition of the profession, guarantee of labor rights, security and protection against various types of violence, as well as respect for the service offered, and the less stigma and prejudice.(1-3)

The situations of vulnerability lived and experienced by them, due to neglect on the part of the State, make it difficult for health professionals to recognize sexuality and sexual health in all their contexts as a human need. It is noteworthy that the term vulnerability can be conceptualized as a condition in which people (such as sex workers) experience, whose situations interfere in the health-disease process, and the confrontation of life becomes impaired as a result of failures in the attention from the State and society.(5) Health professionals, especially nurses, those at the cutting edge of care, focus their care only on preventing sexually transmitted infections (STIs), living aside the subjective issues surrounding sexuality, such as pleasure and sexual satisfaction(5) and subjective and interpersonal conflicts.(1,2) The repressions against sexual service and everything that refers to human sexuality, such as the sexual pleasure and sexual practice of sex workers, are seen by scientific, medical and religious discourses as surrounded by social prohibitions, denials and interventions in the naturalization of sex.(6) The social construction of sexuality occurs through various processes within power relations, among them the power that society exercises over bodies (biopower), determining attitudes consistent with what is expected of boys and girls.(7) 

Sexual satisfaction, as a multifaceted spectrum of sexuality, is conceptualized by the World Health Organization as an indicator of sexual health in the context of quality of life and sexual and reproductive rights, covering issues of physiological (sexual functioning) and also subjective nature under the aegis of affective relationships and the relationship with socioeconomic and cultural factors.(2,8) In this context, the difficulty in recognizing sexuality as a necessity of human life is constant in the practice of nursing professionals, as well as the difficulty to develop proposals that benefit the sexual health of female sex workers, and prevent STIs. These reflections indicate a clear need for nurses to awaken to raise awareness of this problem and then, based on the social representations elaborated by prostitutes about their sexuality, identify meanings that govern attitudes and behaviors, and from this start point, plan practices of specific care for the groups to which these people belong.(8-10) 

The Theory of Social Representations is necessary for studies carried out with vulnerable populations, such as women who work with sexual practice, as it enables the understanding of how these themes are experienced in the daily work of the group, as well as in the way knowledge is elaborated, shared and spread among them.(11,12) In this context, this study aimed to grasp the social representations of sex workers about their sexuality.

## Methods

This is a qualitative study, based on the Theory of Social Representations in its procedural approach. Social representations are instances of practical knowledge that originate in human mental systems (the place where ideas, meanings are built and stored in the unconscious - cognitive system) and that lead to dialogue and to the perception of each person's social, material and ideational context.(11,12) The collaborators of the study were sex workers of the Microregion of Guanambi-BA, headquarters of Alto Sertão Produtivo Baiano - Brazil, which covers 19 municipalities with just over 400 000 inhabitants.(13) The non-probabilistic convenience sample consisted of 69 women who met the following inclusion criteria: aged 18 years and older, and practice of acts of prostitution during the collection period. Since this group has social invisibility, there are few official records about them, either at regional or national level, making it difficult to estimate the population size. The women were contacted through Community Health Agents, who made the invitations in advance and stressed the voluntary and anonymous nature of participation. 

Professionals from the Regional Counseling and Testing Center of Alto Sertão Produtivo municipality approached the participants. Data collection was performed by the coordinator of the umbrella project along with two students (all authors of the present study) who were previously trained to apply the instruments. The instruments were applied between April and June 2017 on an individual base to women who accepted the invitation, in a first stage of the project, in closed rooms of two Basic Family Health Strategy Units and simultaneously by the researchers themselves, located near the workplace of these women. However, as some of them were unable to travel to these units, visits were scheduled, with prior authorization from the Testing and Counseling Center, to collect information at the participants' homes or workplaces. The participants were informed about the project, and its objectives and the reasons/purpose. The researchers introduced themselves, saying their names, and institutional affiliation. Data were produced through the application of a script created by the researchers, which contained items for the characterization of the participants, inducing stimuli for the Free Word Association Test (FWAT), and three open questions to guide the in-depth interview. The questions prepared for characterization of the sample included the variables age, education, religion, job satisfaction, and use of condoms and contraceptive methods. Immediately after that, they said five words that came to mind when they heard the following inducing expressions, one after another: sexual act; sexuality; pleasure. Of the 69 women who contributed to the FWAT, 30 agreed to continue and participate in the in-depth interview, answering the open questions. 

For the in-depth interview, the women answered three open questions: 'Tell me why you associated these words in the previous test'; 'Tell me how you see your sexuality at the moment you are having sex with the client'; 'Tell me about your motivation to continue being a sex worker'. The average time of FWAT responses was 35 seconds for each participant. The interviews were recorded using a mobile device, lasted 15 to 20 min on average, and were later transcribed in full length by typing the speeches in the Microsoft Office Word 2016. The interview made it possible to understand the deepening and the connections established between the evoked words. Data obtained with the FWAT were analyzed by content analysis of words evoked by the constellation target.(14) The analysis took place from the following perspective: comparative analysis of different semantically similar words; determination of internal dualities; organization of words into categories based on frequencies to create a figure with circles where words with higher frequencies (a minimum of 04 was adopted as the cutoff point for frequency of repetition of words) or representative words are found within the circle, and those with lower frequency are left in the periphery.(14) 

Right after that, the speeches from the interviews were analyzed through semantic content analysis,(15) started with a fluctuating reading and followed by a critical reading of the material selected for classification of codes and text units, so as to build inferences and interpretations. Then, thematic, descriptive and qualitative analysis was performed, and allowed the identification of semantic similarities and divergences in the contents of the interpreted and triangulated results, with the result of evocations from the FWAT.(15) 

This work followed the ethical principles of Resolution nº 466/2012 on research with human beings, which was submitted to Brazil Platform for consideration and analysis by the Research Ethics Committee, and was approved with protocol number 2.007.080 / 2017. 

## Results

Of the total participants, the majority were between 18 and 35 years old (78.2%), had a low level of education (53.6%), reported being of the Black race (59.4%), Catholic (55.1%), worked for less than 05 years (68.1%), were not satisfied with their profession (58%), used condoms (63.8%) and contraceptive methods (66.7 %) in sexual relationships. The triangulation of the analysis of the speeches from the FWAT and from the interviews, as well as the convergence by semantic approximation of words, produced two thematic categories: 'Negative representation of sexuality'; 'My pleasure is the money'. These categories reveal aspects arising from the unconscious (region in the brain of people where representations are elaborated, which refers to the cognitive system and symbolic memory) of female sex workers about their sexuality, which are responsible for the formation of meanings, ideas and memory, and therefore constructs of social representations. 

Negative representation of sexuality 

**Figure 1:**
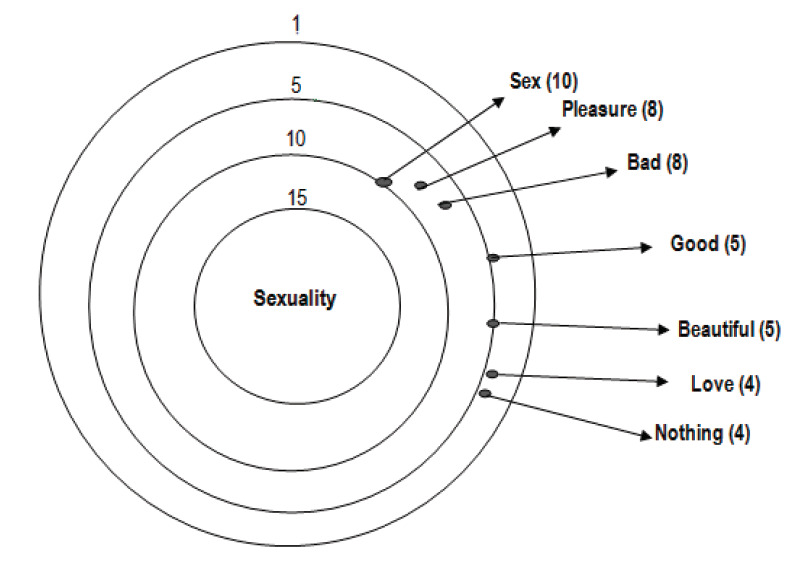
Constellation target of attributes of the stimulus 2: 'Sexuality'

Figure 1 presents the most frequent words (repetitions) evoked by women, and which are statistically significant for the formation of social representations. Thus, the words sex, pleasure and bad stood out, showing how the group represents sexuality, which has its meaning strongly anchored in the sexual practice.

These words are also present in the speeches of the group and express the consensus thinking, showing the acceptance of belonging, as female sex workers and, therefore, the conformation for the construction of their social representations: *I don't know, what do you mean sexuality?* [Sex worker 22]. *When I think of sexuality, I don't think of good things, it's always bad* [Sex worker 3]. *It's not pleasant, it’s disgusting as trash, the only reason why I’m here is to get money, sexuality is very bad, it's really because I need, it’s service* [Sex worker 10]. *I have to show my face, life has ups and downs, I don't give in, I'm afraid of falling in love, afraid of being beaten again, so I worry about finishing quickly* [Sex worker 1]*. Sexuality is sex, in the profession involves money, and in the relationship the feeling, I know how to separate the two moments, it’s different at work, and with my partner* [Sex worker 16]. *It's always bad. I was raped by an uncle of mine when I was a girl, so when any man touches me, I don't feel anything, so I'd rather think about the money, because if it's not because of that, no one man will touch me* [Sex worker 13].

My pleasure is the money

**Figure 2:**
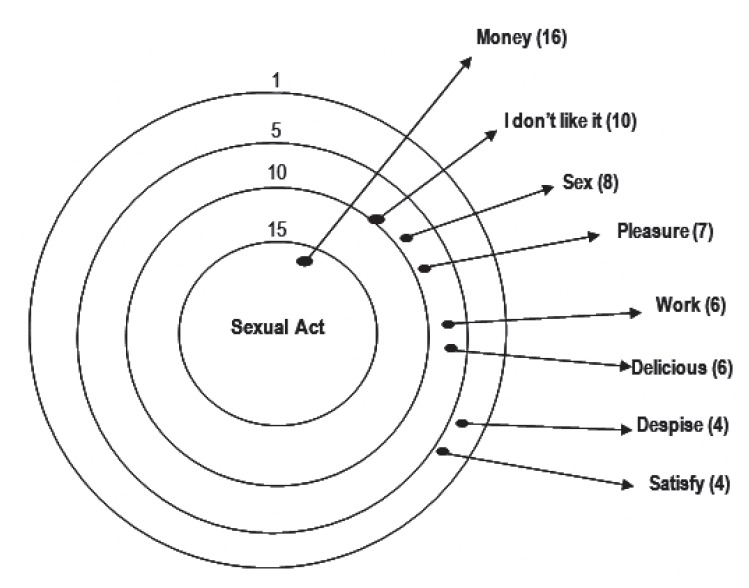
Constellation target of attributes of the stimulus 2: 'Sexual Act'.

**Figure 3:**
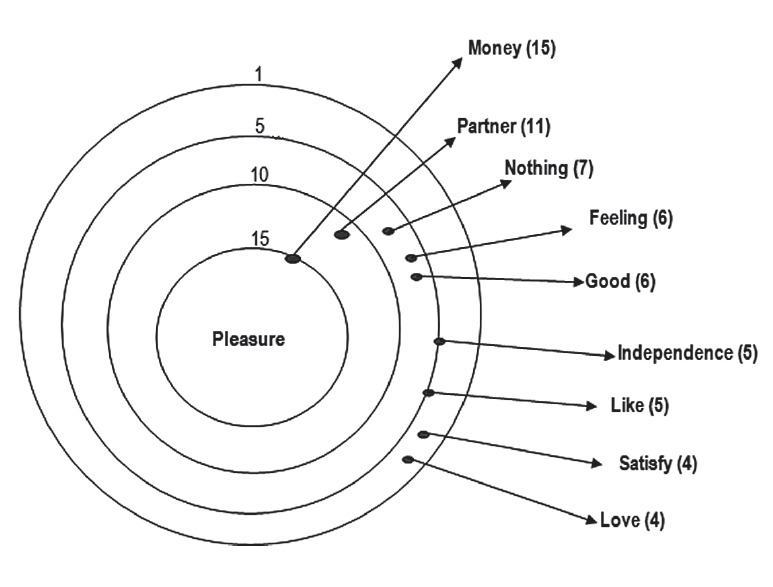
Constellation target of attributes of the stimulus 3: 'Pleasure'.

The evocations that were most significant for the term sexual act were money, I don’t like it, and sex, as can be seen in Figure 2. The inducing term pleasure was associated with money, partner, and nothing, as shown in Figure 3.

The social representations of the group to which these women belong are far from the view of society that sex work alone arouses pleasure and orgasm. It differs from the view presented by the group studied, because they understand that pleasure is associated with feeling of love and affection, and they develop these feeling towards their companions. With the men with whom they practice prostitution, the purpose is to guarantee profit and money for survival, in order to purchase goods and provide quality of life for them and their families. Some even pointed out that when they are not enjoying the moment with the client, they yearn to end the program as soon as possible, as can be seen in the following lines: *When I do a work, I am a professional, I do not choose the man who is with me, I have fun at home with my boyfriend* [Sex worker 19]. *Sex here is for the support of my children, I need to pay my bills, buy clothes. If it wasn't for the needs, I wouldn't make it* [Sex worker 12]. *I can't wait until the end of the month to have money, and a minimum wage doesn't pay my bills! I have even worked in a restaurant, but I feel good here* [Sex worker 7]. *Sometimes the guy doesn't just want to have sex, he comes to talk to me, seek my advice; and I do it, thinking only about money, sometimes I even prefer that* [Sex worker 20]. *At that moment I think about money, what I want for my life, my pleasure is in the money I'm going to earn, and when it's not cool, I close my eyes and just think about money* [Sex worker 9].

## Discussion

It was observed that the social representations of the group to which the women belong in relation to their sexuality are built based on experiences and meanings, where they relate sexuality with the sexual act and the practices that occur in their daily work. Their sexuality in the work environment is expressed in the context of professional practice to meet the needs of both involved (workers and clients). Female workers differentiate their sexual service and the identification (for them) of who their clients are or may be and how their relationships with them should be established. They also determine the duration of work, the amount, and the forms of payment. Thus, relationships and pleasure in the sphere of sexuality are determined by the sex workers, where the payment of money represents the way the relationship is represented, both to the provider (prostitutes) and to the consumer (clients).(16) In this context, the practice is carried out without guilt by some (those who are empowered and have faced adversity in their journey in this profession and have taken the control of situations), but qualified as negative, bad, not pleasant by others, due to the vulnerabilities to which these labor practices expose them, arising from social stigmas and from the absence/lack of state protection, such as fear of violence.(17) 

It is reiterated that human sexuality and, within its context, sexual health, has been a challenge for groups attached to social stigmas and vulnerable populations. This makes it important to understand this phenomenon and to raise hypotheses about the representational centrality of sex workers about their sexual satisfaction.(18-20) Social representations are elaborated based on their understanding of pleasure and the way it is felt and experienced with clients or partners in some cases. However, sexual satisfaction had a negative connotation in most cases. Sex workers set symbolic limits on their own bodies as to personal and professional life, based on what they determine that can or cannot be done sexual intercourse. Within these limits, it is the sexual satisfaction associated with orgasm (pleasure), because satisfaction is experienced either in the private space with the partner or by a client who aroused in them some feeling (personal life). The professional relationship developed with other clients, whose purpose is to make them achieve pleasure and obtain the established profit, refers to their professional life and the 'sexual contract' signed with men.(21) 

However, by having access to goods and meeting needs, the pleasure is represented by the profit obtained from sex work(22) also presented in a study carried out in France with French prostitutes, problematizing the fact that society considers the exchange of sexual pleasure for swearing of masters and fantasies acceptable, but cannot value sex and charge the satisfaction reached with it.(23) It is in sex that money becomes synonymous for lust, love, leisure, and fun, not as a tool for buying or exchanging, but as an object of desire and sexual exchange.(24) 

In a survey of 30 female sex workers in the city of Barcelona, Spain, the authors showed, based on the experience of the participants, that addressing the experiences of prostitution transcends environmental and personal concerns.(6) They are structural problems where sexuality is related to bad experiences of professional practice, and reflect the interpenetration of these structures in the individuals’ lives, both in their social and historical context, with the association of denial with orgasmic pleasure in the sexual act with clients.(6) In another study of 40 female sex workers aged 15 to 25 in Cabo Verde, Rio Grande do Sul, it was identified that these women seek their partners, even in the practice of prostitution, not just the desire, which is often not found, but also economic means to support the family. They point out that orgasm is not sustained in this type of relationship, because they associate the practice with a profession.(25) This representation of the relationship with the client possibly originates in childhood, on the part of some, due to acquired vulnerabilities such as exposure to promiscuity and indirect incentive to prostitution; however, it is emphasized that most of them are in sex work of its own accord, because they see that this profession makes it possible to have access to goods and services that are often neglected by the State, escaping the stereotype created in society that sex workers have no other choice of profession; indeed many have, however the subhuman conditions proposed by employers are even an affront to their needs.(26) 

The representational consensus of sexuality, whether due to profit and negation of sexual pleasure, grasped from the collective memory of sex workers, is associated with the sexual act and is in line with the representational discourses of sex workers in the bohemian zone located in the center of Belo Horizonte.(27) However, as noted in the positive elements such as self-esteem, good, affection, vibe, and feeling, even though orgasm itself is rare in the case of clients, studies suggest that it may occur when there is a greater bond between prostitutes and steady partners or clients with whom they develop a more affective relationship.(1,28) This view is in line with the thinking of feminists who have embraced the concept of empowerment and undo discourses of oppression of victims and women's lack of resilience. Thus, these discussions assume an essential role for the contextualized understanding of prostitution as a social construct, both for the analysis of the constitution of micro-powers in the production of discourses on sexuality and subjugation of women, who have their sexuality and their body exposed as a bargaining chip(7) as in social representations(12) which are built by the experience as a group and by common sense, which interfere with behaviors.

Regarding the effects, this type of stigmatization shows that the way they experience these issues in their daily work interferes with the knowledge of sexuality, contrary to what the scientific community thinks as they mean, especially because they have not discovered or awaken in themselves and reverberate in the depictions of sexuality alluding to profit.(29) To understand the division between the professional and the personal universe, it is necessary to understand the unit of professional activity that these women call the 'date', that is, an elementary unit of the activity of sex workers. Its performance requires agreement on three issues: the inherent work practices, the amount to be charged, and the time of the activity. These practices refer to both the paid sexual act, and to the time the professional spends with the client to talk, without performing any activity considered sexual. And this is what differentiates their sexuality from their professional life, as well as that related to their affective life.(29-30)^.^

Although invisibilities exist, men perceive these women as 'sexually depraved', and therefore seek them to meet their needs, because 'normal' wives or women cannot satisfy them, reaffirming the Judeo-Christian theory of sexual satisfaction associated with a sinful and deviant act.(26) Therefore, specifically in this study, the pleasure aroused by money and not by the partner was a unanimous thinking in the social representations of these women, which reveal facets and nuances of sexuality of this group of women, beyond what macho society establishes as a body to be used for a purely sexual purpose. The speeches of women showed particularities in relation to their work, according to the representations learned about the source of pleasure during the sexual act: the body represented as a work object to obtain money. The body and its borders are legitimizing objects of relationships and partnerships established both inside and outside prostitution; they transversalize the exclusively sexual function to build social meanings of being in the public and private world.(30) 

The practice of prostitution contributes to the formation of belonging of a group loaded with social stigmas, which have in their cognition systems representations about sexuality that are distinct from those of society and, specifically, by experiencing sex as work***.*** Sexuality is a multifaceted subject full of contradictions, as it involves feelings, senses and meanings. No health professional can remain indifferent. In order to reduce the exclusion and prejudice associated with prostitution on the part of health professionals and health sectors that provide this type of service to the population, it is essential that scientific studies produce differences in the way they act, think, feel and believe of the particularities associated with this practice.(9,26) 

It is concluded that the social representations elaborated by the sex workers of this study reveal the way these women experience sexuality and the rent of their bodies in their daily work, as well as the meanings seized/perceived in the mental fields (unconscious). The meaning of sexuality is linked to the sexual act practiced with clients as a source of income and profit to obtain goods and supply their needs. However, pleasure toward sexual satisfaction is anchored in affection and emotions developed with steady partners, or negative feelings when associated with sex with a client. 

The dispute between subjective (pleasure) and practical (obtaining income) meanings of daily prostitution helps to understand the importance that the meanings attributed by sex professionals to the type of work they perform influences the constitution and formation of representations about sexuality, specifically the association with money. 

The contribution of this study lies in the fact that nursing professionals, especially nurses, may focus their attention and practices on the meanings of sexuality originated in the social representations expressed by sex workers. By doing so, they will be able to rethink their care strategies for the sexual health of this group of women, encompassing not only the prevention of STIs but also the subjectivity that surrounds sexuality, such as sexual satisfaction, pleasure, emotions and imbricated feelings. As a result of their (sexual) work, these women are stigmatized and socially marginalized, face shame and institutional prejudice, and therefore do not access health services.

This study had as limitations the number of women and the fact that it took place in a poor area in northeastern Brazil, which makes completely prevents generalizations because women and people, in general, have different profiles and living conditions, varying according to culture and place. However, the importance of expanding studies on the health of female sex workers, especially of qualitative nature, is stressed because the daily routines and experiences of these women vary. Such studies may contribute to the appreciation of different contexts, meanings and representations. 
